# Scaling-up digital follow-up care services: collaborative development and implementation of Remote Patient Monitoring pilot initiatives to increase access to follow-up care

**DOI:** 10.3389/fdgth.2022.1006447

**Published:** 2022-12-07

**Authors:** Salomé Azevedo, Federico Guede-Fernández, Francisco von Hafe, Pedro Dias, Inês Lopes, Nuno Cardoso, Pedro Coelho, Jorge Santos, José Fragata, Clara Vital, Helena Semedo, Ana Gualdino, Ana Londral

**Affiliations:** ^1^ Value for Health CoLAB, Lisbon, Portugal; ^2^ Comprehensive Health Research Center, Nova Medical School, Nova University of Lisbon, Lisbon, Portugal; ^3^ CEG-IST, Instituto Superior Técnico, University of Lisbon, Lisbon, Portugal; ^4^ LIBPhys (Laboratory for Instrumentation, Biomedical Engineering and Radiation Physics), NOVA School of Science and Technology, Campus de Caparica, Caparica, Portugal; ^5^ Fraunhofer Portugal AICOS, Porto, Portugal; ^6^ Hospital de Santa Marta, Centro Hospitalar Universitário Lisboa Central, Lisbon, Portugal

**Keywords:** collaboration, follow-up care, implementation, participatory action research, remote patient monitoring

## Abstract

**Background:**

COVID-19 increased the demand for Remote Patient Monitoring (RPM) services as a rapid solution for safe patient follow-up in a lockdown context. Time and resource constraints resulted in unplanned scaled-up RPM pilot initiatives posing risks to the access and quality of care. Scalability and rapid implementation of RPM services require social change and active collaboration between stakeholders. Therefore, a participatory action research (PAR) approach is needed to support the collaborative development of the technological component while simultaneously implementing and evaluating the RPM service through critical action-reflection cycles.

**Objective:**

This study aims to demonstrate how PAR can be used to guide the scalability design of RPM pilot initiatives and the implementation of RPM-based follow-up services.

**Methods:**

Using a case study strategy, we described the PAR team’s (nurses, physicians, developers, and researchers) activities within and across the four phases of the research process (problem definition, planning, action, and reflection). Team meetings were analyzed through content analysis and descriptive statistics. The PAR team selected ex-ante pilot initiatives to reflect upon features feedback and participatory level assessment. Pilot initiatives were investigated using semi-structured interviews transcribed and coded into themes following the principles of grounded theory and pilot meetings minutes and reports through content analysis. The PAR team used the MoSCoW prioritization method to define the set of features and descriptive statistics to reflect on the performance of the PAR approach.

**Results:**

The approach involved two action-reflection cycles. From the 15 features identified, the team classified 11 as must-haves in the scaled-up version. The participation was similar among researchers (52.9%), developers (47.5%), and physicians (46.7%), who focused on suggesting and planning actions. Nurses with the lowest participation (5.8%) focused on knowledge sharing and generation. The top three meeting outcomes were: improved research and development system (35.0%), socio-technical-economic constraints characterization (25.2%), and understanding of end-user technology utilization (22.0%).

**Conclusion:**

The scalability and implementation of RPM services must consider contextual factors, such as individuals’ and organizations’ interests and needs. The PAR approach supports simultaneously designing, developing, testing, and evaluating the RPM technological features, in a real-world context, with the participation of healthcare professionals, developers, and researchers.

## 1. Introduction

The last two decades of research and development (R&D) on remote patient monitoring (RPM) technologies mainly focused on the technicalities of providing care in unusual environments (1). Although RPM pilot design changes on a case-by-case basis, the system should comprise three components: the care team organization, the technology used, and patient engagement activities (2). Several pilot initiatives emerged from chronic diseases (3) to episodic care [e.g., surgical follow-ups (1)] to exploit the benefits of RPM, such as, continuity of outpatient care (4), improved healthcare decision-making (1), complications anticipation (4), and cost reduction (5). In surgical follow-up care, RPM can significantly reduce severe complications and increase patient safety in the most critical period after hospital discharge (6) by supporting healthcare professionals in the continuous surveillance of clinical and patient-reported outcome measures (PROMs). However, research on implementation is scarce (7). In addition, after coronavirus disease 2019 (COVID-19) pandemic outbreak, the increasing demand for RPM-based services to cope with postponing surgeries (8) and shifting in-person to remote care (9) drove ongoing pilot initiatives to scale up faster without planning (10). Constrained by time and resources, the design, development, and implementation of the scaled-up services pose risks to the access and quality of care (11).

After COVID-19, some peer-reviewed work focused on RPM implementation and two generated relevant knowledge (2,12). A French case study documents the design methodology used by a multidisciplinary team for an RPM system for cancer care (2). The authors position the RPM implementation as both a technological and organizational innovation. Therefore, an RPM service is a socio-technical system framed within a local context. The authors also reinforce the collaboration between multidisciplinary groups for proper needs identification. In a rapid implementation of a COVID-19 RPM intervention in the United States of America (USA), a team with industry, healthcare delivery, and academia members repurposed an existing application. Their study revealed an increased patient perception of safety and the emergence of an organizational method of resident and staff rotation (12). Nevertheless, both works are context-specific and different from the surgical follow-up context, which is this paper’s focus. On top of that, the first work lacks deeper knowledge of how the design, implementation, and evaluation phases are related. The second work had limitations on feature customization, reducing the opportunity to absorb feedback through iterative development cycles.

Scalability and rapid implementation of RPM services require social change and active collaboration between stakeholders. Therefore, a participatory action research (PAR) approach is needed to support the collaborative development of the technological component while simultaneously implementing and evaluating the RPM service through critical action-reflection cycles (13). Throughout a research process characterized by nonlinear, recursive cycles of action and reflection, the PAR team characterizes problems in specific contexts, understands the required changes in socio-technical systems, and consequently defines, implements, and evaluates actions to improve practice (14). There are several expected outcomes from following a PAR approach. For the scope of this paper, the farmer participatory research model (15) highlights some of the most relevant outcomes that can be adapted for the implementation of RPM services: (1) generation and adoption of new appropriate technologies by healthcare professionals and patients to increase RPM uptake in surgical follow-up; (2) better understanding, on the part of researchers, of the existing surgical follow-up service; (3) scientific characterization and understanding of the socio-technical-economics constraints to sustainable RPM care delivery; (4) development of RPM technologies that meet patients and healthcare professionals needs; (5) improved research and technological systems as a consequence of a close collaboration; (6) empowerment by improving healthcare professionals’ capacity for self-directed technology development; and finally (7) ability to adapt healthcare systems to changing conditions.

In this paper, we propose a PAR approach to guide researchers, developers, and healthcare professionals in scaling up RPM pilot initiatives and implementing RPM-based surgical follow-up services. Based on a case study in the cardiothoracic surgical department, in a Portuguese public hospital, we analysed feedback from interviews, reports, and meeting minutes from different stakeholders in the context of three previous different RPM pilot initiatives, to identify and prioritize the features required to efficiently scale up an RPM-based system.

## 2. Methods

### 2.1. Context, procedure, and participants

#### 2.1.1. Context

In 2020, in the scope of the COVID-19 pandemic, the Portuguese Foundation for Science and Technology (Fundação Portuguesa para a Ciência e Tecnologia - FCT) launched a tender to support Research and Development (R&D) projects in the areas of data science and artificial intelligence (AI) in Public Administration (16). The main objective was to promote projects that could cope with pandemic-imposed challenges, improve public health services, and support citizens in better decision-making concerning health behaviors. FCT required the participation of at least one public administration entity providing health care committed to using the project results and the R&D activities. Another requirement was to provide a Data Management Plan that preserved the use of data ethical and legal aspects, such as privacy and consent issues in citizens’ data access, data sharing across different sources, and transparency of the analysis and utilization. The projects could last 24 to 36 months with a maximum funding limit per project of 240 thousand euros. This tender allocated 3 million euros from a national-based fund budget.

A consortium of four partners working with RPM technologies received approval and funding for a 36-month project, evaluated with 7.3 on a 10-point scale as a “High-Impact” proposal. With a total budget of approximately 240 thousand euros, the main goal was to leverage an ongoing RPM-based follow-up pilot service at the Cardiothoracic Surgery Department of Hospital de Santa Marta, Lisbon, Portugal. The challenge involved consolidating each partner’s previous work with RPM technologies to make the system more robust and ready to integrate intelligent and adaptable digital tools that could allow the redesign and value assessment of the RPM surgical follow-up service for at least 150 patients.

#### 2.1.2. Procedure

The four partners, familiar with the PAR approach and respective expected outcomes, agreed to conduct this type of research to define and prioritize the development of the RPM platform features. One of the most relevant reasons is its application as a bottom-up research and development strategy for technology transfer (15,17,18) within the hospital walls. More specifically, the PAR approach is very relevant when trying to improve real-world practices because it involves a research team (PAR team) that includes researchers and practitioners who contribute actively with their scientific and practical knowledge in all research procedure phases (15).

This paper describes, using a case study strategy, how and why the PAR team identified features from previous projects and prioritized the development of an enhanced RPM-based platform in a real-world setting when there was no control over contemporary events (19). The case study has been referred to as an appropriate research strategy to enhance the phases and the transitions across the reflection and action phases (19,20). This strategy becomes particularly useful in health services research (21), particularly in this paper, to better evaluate the essential RPM-based follow-up services’ features to each relevant stakeholder and why in the context of cardiothoracic surgery (22).

**Figure 1:**
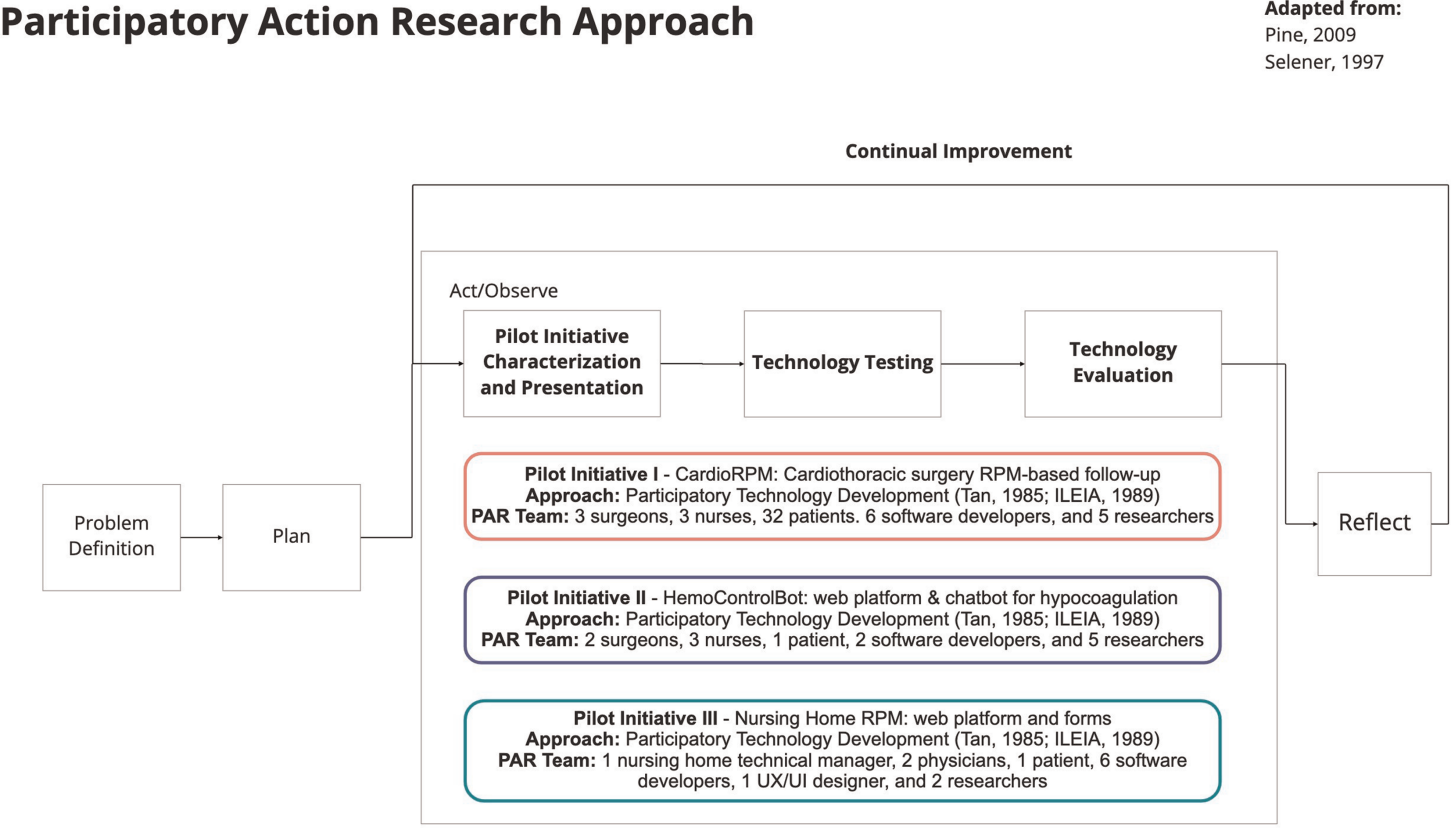
Participatory research process adapted from Selener (15) and Pine (18).

The participatory research process presented in Figure 1 involved four phases that lasted 27 months. The process is an adaption from the cycle described by Pine (18) and the models for Participatory Action Research in Organizations and the Farmer Participatory Research described by Selener (15). In the following sections, we describe the activities conducted in each phase.

#### 2.1.3. Participants

The four partners were categorized into two groups according to the type of organization:

• **Hospital:** Hospital de Santa Marta is a state-owned public Central Hospital serving the Greater Lisbon area as part of the Central Lisbon University Hospital Centre (CHULC). It is one of the Country’s leading Internal Medicine schools and one of Portugal’s main reference centers for diagnosing and treating cardiovascular disease (23).
• **Research Laboratories:** One of the research laboratories focuses on applied and project-oriented research in technology development for predictive, preventive, personalized, and participatory medicine (24). The second research laboratory focuses on supporting, developing, and fostering clinical, public health, and health services research (25). The third research laboratory focuses on validating innovative methodologies to measure outcomes and costs and provide trustful scientific evidence under Value-based Healthcare principles (26).

Each partner was responsible for selecting the PAR team participants and classifying the members’ roles according to their interests in the research process without requiring a minimum number of participants. The team was composed of 12 members split according to three roles types (act as a proxy for the stakeholder groups):

• **Researchers:** the main interest of this group was to conduct research to generate and contribute knowledge to their scientific area. The research laboratories selected six researchers based on their expertise in digital health services design, digital health literacy, cost analysis, and previous relationship with the healthcare professionals team.
• **Developers:** the main interests of the developers include designing, programming, building, deploying, and maintaining features through the use of different skills and programming tools. The research laboratories selected six software developers based on their skills in AI and software development and previous relationship with the healthcare professionals team.
• **Healthcare professionals (end-users):** the main interest of healthcare professionals was to use technology to enhance their performance and provide high-quality patient care. In the context of the proposed technological solution, the end-users are physicians and nurses of the Cardiothoracic Surgery service selected by the service’s director. Therefore, the PAR team included three physicians and three nurses to collaborate with the developers and researchers to solve RPM-related problems. They were selected based on their previous relationship with researchers and developers and expertise in RPM-based follow-up services.

### 2.2. Problem definition phase

The Problem Definition Phase aimed to clarify the problem and involved three activities at two different moments: the first at the time of the grant proposal writing and the second, in a 90-min video conference group meeting, after the grant award notification. The activities for the problem formulation are the ones suggested in Design Science Research: precise problem definition, problem positioning and justification, and root causes identification (27).

The grant proposal writing included two activities. First, the PAR team conducted a literature review of academic publications (fields of cardiothoracic surgery, AI and data science, and decision support systems engineering). The team defined the problem through online collaboration using a web-based word processing application because it allows keeping track of changes and comments. To formulate the problem more precisely, the team used the web application to leave comments with feedback on others’ contributions. The final version of the problem was: in the COVID-19 pandemic context, characterized by a high demand for remote care to avoid infection, there is limited access to RPM-based surgical follow-up services.

The second activity aimed to position and justify the problem so the different PAR groups could engage in the proposal writing. Therefore, each group consolidated their perceived reasons for the problem to be relevant. The PAR team’s opinions of why the problem is significant and to whom can be split into two stakeholder groups: (i) Healthcare professionals and providers and (ii) Software developers and Researchers. On the one hand, the first group contextualized the problem as an obstacle to providing treatment to a larger sample of patients while maintaining essential high-quality healthcare using RPM and transferring the acquired knowledge and technology to other surgical follow-up services. *“(Senior Surgeon) stressed that the objectives are to continue and expand the monitoring of patients undergoing cardiothoracic surgery and that this monitoring can be extrapolated to other units.”*. On the other hand, the second group positioned the problem considering its scalability and the need to implement a clinical study with real-world evidence to analyze the costs and benefits of a generalized RPM service. *“(Senior researcher) mentioned that it could be interesting in making the RPM platform available for other problems.”*

After the grant award notification, during the project kick-off session, the PAR team focused on finding the root causes of the problem. The meeting was planned to last one hour, and all PAR team members were invited. Due to COVID-19 constraints, the meeting occurred via a video-based communication platform. To guarantee that the researchers were seen as colleagues, there was no moderator (15). There was only a note-taker, which was randomly assigned at the beginning of the meeting. The note-taker (27) wrote down all mentioned root causes, which were analyzed by the researchers using content analysis (28). Finally, the root causes were represented using an Ishikawa diagram (29). Additional details on the root causes and respective categories of the defined problem are described in the Ishikawa diagram (Supplementary Material 3).

### 2.3. Planning phase

As mentioned in the context subsection, the PAR team had to identify features from previous projects and prioritize the development of a more robust RPM-based platform capable of integrating AI-based and value assessment tools. Therefore, the planning phase aimed at promoting the discussion on the required actions to reach two objectives: (i) decide which pilot initiatives should be investigated for feature extraction; (ii) define the data collection and analysis plan for features’ characterization, evaluation, and prioritization, and reflection on the PAR approach performance.

The PAR team planned one 60-min video conference meeting to achieve these goals. The study selection was based on three inclusion criteria: the project followed a participatory approach, involving at least one of the four partners, who had the ownership of the intellectual property rights.

The researchers used Table 1 to guide the rest of the team in deciding on the appropriate data sources and collection and analysis methods to answer the research questions associated with each pilot initiative characterization. In this phase, 10 PAR team members met in one meeting, which lasted approximately one hour. The two main results of this phase are: the list of pilot initiative studies to extract features and the data collection and analysis plan to characterize each pilot initiative and respective features (Table 2); and the data collection and analysis plan to evaluate and prioritize each feature and reflect on the PAR approach performance.

**Table 1:** Research questions and the respective data collection and analysis methods.

Research question (RQ)	Data collection methods	Data analysis methods	Literature examples
RQ_1 - What are the most important features of RPM-based follow-up services for patients?	Interviews, Reports	Transcription and Grounded Theory, Content Analysis	(30)
RQ_2 - What are the most important features of RPM-based follow-up services for healthcare professionals?	Meeting minutes, Reports	Content Analysis	(31)
RQ_3 - What are the topics and focus of the interactions among PAR team members while defining the requirements and features for a new solution?	Meeting minutes PAR team data	Content Analysis, Descriptive Statistics	(32)
RQ_4 - What is the type and frequency of contribution from each PAR team role?	Meeting minutes, PAR team data	Content Analysis, Descriptive Statistics	(27)
RQ_5 - How requirements and features definition is described by the PAR iterative cycle?	Meeting minutes	Content Analysis	(15)

**Table 2:** List of pilot initiatives and respective data collection and analysis plan.

Pilot initiative	Data collection methods (data analysis methods)
1 - CardioRPM: Patient follow-up after cardiothoracic surgery	Video-recorded in-person semi-structured interviews (grounded theory)
	Meeting minutes (content analysis)
	Reports (grounded theory)
	PAR team data (descriptive statistics)
2 - HemoControlBot: Oral anticoagulation therapy management	Phone call-based semi-structured interviews (grounded theory)
	Meeting minutes (content analysis)
	PAR team data (descriptive statistics)
3 - NursingHomeRPM: Remote care delivery in nursing homes	Meeting minutes (content analysis)
	Reports (grounded theory)
	PAR team data (descriptive statistics)

The PAR team decided that for each pilot initiative, the features should be presented, tested, and evaluated in web-based or in-person (hospital visits) group meetings. They also agreed to use meeting minutes to report participants’ observations and to use content analysis to support the decision of the final list of features and the evaluation of the PAR approach using content analysis (Supplementary Material 1). The format of the group meeting was also a result of the planning phase. At the beginning of each session, the researcher responsible for writing the minute asked for the consent of the participants to take notes on the interventions made by the participants and their respective reactions to the team. At the end of the meeting, the researcher read the out loud minute, requested approval, and proposed a date and agenda for the following meeting.

### 2.4. Action/observation phase

This phase involved two main activities through several not pre-planned group meetings: (i) the researchers characterized and presented the pilot initiative, its resulting features, and respective end-users feedback evaluation; and (ii) the PAR team tested and evaluated the features.

In the first activity, the researchers described the pilot initiative goal, deployment site, targeted population, previous and new intervention, RPM period, RPM collected outcomes, responsible team, the technology used, pilot activities, pilot duration, end-users involved, and end-users feedback data collection methods. For each identified feature, the researchers presented its originator, and end-users’ feedback. This information was obtained through content analysis of internal and external reports and minutes, and grounded theory analysis of interview transcripts. Internal and external reports were used to record activities execution, bugs and issues identified, and consolidate generated knowledge. Two researchers read each report, extracted all the relevant information, and combined it in a spreadsheet. For each information extracted, the researcher recorded the report date, author, and role. Similarly, all minutes’ observations were copied to spreadsheets, one spreadsheet per meeting. For each observation extracted, the researcher recorded the author. As defined in the planning phase, minutes and reports were coded according to the categories (topic, focus, interaction, reaction, outcome, and agent) described in Supplementary Material 1.

In two pilot initiatives, semi-structured interviews were used to collect patient emotions, attitudes, opinions, and experiences through two different formats: video recording and phone calls. For both, there was one researcher that transcribed each interview in Portuguese. The grounded-theory method was used and included three rounds (33). In the first round, two researchers coded the interviewer’s and interviewee’s quotes as units of text to identify the most frequently covered themes. The researchers combined the emerging themes (feedback on existing features, improvements and new ideas, generated learning, proof of acceptance, and reflections) according to their similarity and deleted the duplicates. The output of the second round allowed the researchers to define a list of possible values for each theme. The third and final round allowed the researchers to code each unit of analysis according to Supplementary Material 2. In the three rounds, when disagreements occurred, the authors reached a consensus via discussion.

The PAR team tested and evaluated the features in the second and final activity. For each feature, the team assessed the required effort, type of changes, and value for the end-user. The format and analysis of the group meetings followed the configuration and data analysis methods agreed on during the planning phase. It is crucial to notice that the presentation, testing and evaluation occurred cyclically per feature or set of features. However, we present the features’ evaluation per pilot initiative to demonstrate the results better.

### 2.5. Reflection phase

The reflection phase involved several not pre-planned group meetings to define the final set of features and prioritize the development based on reflections on (i) the pilot initiative participatory level assessment; and (ii) the team’s evaluation of each feature. The participatory level assessment in each pilot initiative consisted of descriptive statistics of the type and level of participation and representation of each stakeholder group. The team used the MoSCoW (must have, should have, could have, and will not have this time) prioritization method (34) to help define agile and fast development sprints based on the previous reflections (35). When disagreements occurred, the PAR team reached an agreement via discussion, considering time and budget constraints and expected outcomes. Finally, the team reflected on the PAR approach performance through descriptive statistics of the meeting minutes content analysis concerning the number of participants and contributions made per phase, focus, topic, and research outcome more frequently referred per phase.

## 3. Results

The next subsections describe the results obtained in the two main phases of the PAR approach in the context of this work: action and reflection.

### 3.1. Action/observation phase

This phase involved 11 meetings with an average participation of nine PAR team members and a duration of 44 min (8 h and 5 min in total). Each pilot initiative followed an iterative development approach illustrated in Supplementary Material 4, the corresponding study was approved by each deployment site’s ethical committee, and all participants signed informed consent.

The first pilot initiative refers to the RPM follow-up pilot service that the PAR team proposed to leverage in the scope of the funded project (Box 1). Therefore, the motivation, goal, deployment site, digitization model, and groups of end-users are the same. The main difference is the new project’s requirement of covering at least 150 patients.

Box 1. **Pilot Initiative 1: CardioRPM: Patient follow-up after cardiothoracic surgery (36)**

**Motivation:** The healthcare professionals from the cardiothoracic surgery needed to automatize health-related outcomes collection during the follow-up period after cardiothoracic surgery.

**Goal:** Digitize the follow-up service of patients after cardiothoracic surgery.

**Deployment Site:** Hospital de Santa Marta, Lisbon, Portugal.

**Population:** Cardiothoracic surgery patients.

**Previous intervention:** The standard follow-up version of this service consisted of phone calls to the patient at three days, one month, three months, six months, and 12 months after the hospital discharge.

**New intervention:** The responsible surgical team proposed the integration of IoT devices to monitor patients remotely in the comfort of their homes during the first month after surgery to detect problems and avoid acute problems early.

**RPM Period:** 30 days.

**RPM Outcomes Measurements:** 11 in total: blood pressure and heart rate, weight, number of steps, the occurrence of blackouts, perceived alterations in surgical wound healing, picture of the surgical wound, presence of palpitations, presence of edemas, presence of dyspnea, chest pain intensity level.

**Responsible Team:**

Nurses: total 3 (3 female, average age of 48±4.9 years old);

Physicians: total 3 (2 male, average age of 49.5±18.5 years old);

Developers: total 4 (1 female and 3 male, average age of 29.5±3.6 years old);

Researchers: total 5 (4 female and 1 male, average age of 35.2±10.4 years old) with expertise in digital health services design, digital health literacy, and cost analysis.

**Pilot activities:** The surgical team agreed with the existing version of the IoT kit, which includes a weight scale, blood pressure monitor, smart wristband, and an Android smartphone. A mobile application allowed patients to report outcomes through four (Yes/No) questions survey and two 4-point Likert scale questions, and a smartphone camera, and collect automatically clinical parameters from the IoT devices.

**Pilot duration:** The mobile application and the IoT devices’ main development was conducted in the context of heart failure and served as a basis for a customized version for this pilot. The team worked together to create an RPM platform that could ensure interoperability with the existing mobile application and IoT devices.

**Pilot Duration:** February 2019 to January 2021 (22 months).

**End-users involved:**

Patients: total 35 (18 male and 17 female, average age of 59.9±13.4 years old);

Nurses: total 24 (2 male and 22 female);

Physicians: total 3 (3 male).

**End-users feedback data collection methods:** On the last day of the follow-up period, the patient replied to a service satisfaction survey and provided feedback on the overall experience through a video-recorded semi-structured interview.

The pilot initiative 1 characterization resulted in nine features (Table 3) which are: (1) outcome collection using a mobile app connected to IoT devices; (2) outcome collection using smartphone camera; (3) RPM-based therapy management; (4) web-based RPM care management platform; (5) outcome-based automated alerts; (6) RPM dynamic table; (7) RPM activities management and resource allocation monitoring; (8) an integrated ticket reporting system; and (9) periodic data fetching. The PAR team evaluated the first eight providing the most value for the end-users (patients, nurses, and physicians) since the patient, nurse, and physician feedback was very positive. Finally, the ninth feature was excluded because there was a need to have data available more frequently. The features of outcome collection using a smartphone camera, web-based RPM care management platform, and RPM activities management and resource allocation monitoring were evaluated has requiring major changes and, consequently, high effort, because of the integration of AI-based tools for optimal follow-up resources prediction based on patient risk stratification.

**Table 3:** List of features of pilot initiative 1.

Feature (originator)	Utilization feedback	HP feedback (N)	Patient feedback (N)	PAR team evaluation
1 - Outcome collection using a mobile app connected to IoT devices (Physicians)	On average, a patient answered 92.9% of the questionnaires.	Suggestion to add a new PROM question (1); Suggest that answers should cope with intermediate improvements (4); Suggest improvements to instructions (2);	Patient/caregiver share that measuring the outcomes were part of his/her daily routine (8); Good, but found some problems/challenges (12); Did not like it or could not use it, due to problems (2) Suggest that answers should cope with intermediate improvements (3); Suggest improvements to instructions (2)	Minor changes, low effort, high value
2 - Outcome collection using smartphone camera (Nurses)	Average number of pictures sent from the patient is 44.	Good, but found some problems/challenges (2)	Good, but found some problems/challenges (6); Did not like it or could not use it, due to problems (3)	Major changes, high effort, high value
3 - RPM-based therapy management (PAR team)	Total number of messages sent to patients: 300	Suggestion to add a new feature (1)	No feedback recorded	Minor changes, low effort, high value
4 - Web-based RPM care management platform (PAR team)	27 registered accounts; average session time of 9 min and 45 s	Suggest improvements (6)	Patient/Caregiver highlights the perceived support he/she got from the HCP (13); Patient/Caregiver recommends the RPM service to other patients (12); Having access to high-quality remote care delivery at the comfort of home (10)	Major changes, high effort, high value
5 - Outcome-based automated alerts (Nurses)	No metric recorded	Suggest improvements (6)	Having access to high-quality remote care delivery at the comfort of home (10); Patient/Caregiver highlights trusting in the system because of HCP calls (9); Being able to recover in a safe and friendly environment (8)	Minor changes, low effort, high value
6 - RPM dynamic table (Nurses; Developers)	No metric recorded	Suggest improvements (2)	No feedback recorded	Minor changes, low effort, high value
7 - RPM activities management and resource allocation monitoring (Physicians; Researcher; Developers)	Total number of clinical actions and notes reported: 242	Suggest improvements (2)	No feedback recorded	Major changes, high effort, high value
8 - An integrated ticket reporting system (Developers)	0 tickets recorded	No feedback recorded	No feedback recorded	Minor changes, low effort, high value
9 - Periodic data fetching (Developers)	No metric recorded	No feedback recorded	No feedback recorded	Excluded

The second pilot initiative refers to another RPM follow-up pilot service in the same hospital and surgical department as the funded project (Box 2). This case was selected because part of the population submitted to cardiothoracic surgery might require oral anticoagulation therapy in the long term. Therefore, understanding the technological features to provide continuous care to these patients is relevant.

Box 2. **Pilot Initiative 2: HemoControlBot: Oral anticoagulation therapy management (37)**

**Motivation:** A private medical device company needed to demonstrate the added value of the coagulometer when integrated into an RPM service.

**Goal:** Digitize the oral anticoagulation therapy management after cardiac surgery.

**Deployment Site:** Hospital de Santa Marta, Lisbon, Portugal.

**Population:** Patients under oral anticoagulation therapy after cardiac surgery.

**Previous Intervention:** The standard oral anticoagulation therapy involves frequent patient visits to the hospital to measure the International Normalized Ratio (INR) value to assess the blood’s ability to clot. Based on this and other relevant outcomes, the physician adjusts medication to avoid the risk of bleeding.

**New Intervention:** The responsible surgical team and a private medical device company proposed the combination of a coagulometer and a mobile text message-based RPM system to allow patients to report remotely therapy-relevant outcomes.

**RPM Period:** 180 days.

**RPM Outcomes Measurement:** 7 in total: INR, the dosage of antibiotic and anti-inflammatory drugs, the occurrence of bruises, hemorrhages, feces, nausea, and the number of trips to the hospital or health center.

**Responsible Team:**

Nurses: total 3 (3 female, average age of 42±10.5 years old);

Physicians: total 2 (2 male, average age of 42±15.6 years old);

Developers: total 2 (2 male, average age of 26±1.4 years old);

Researchers: total 5 (2 male, average age of 36±11.6 years old) with expertise in digital health services design and cost analysis.

**The technology used:** The surgical team demanded that the RPM system should not be dependent on the type of mobile phone to increase accessibility. Therefore, patients reported the outcomes by answering seven 4-point Likert scale questions via the lowest cost communication channel, i.e., short message service (SMS).

**Pilot Activities:** The team worked together to create an SMS-based RPM platform that automatically generates SMSs asking the patient to report the required outcomes.

**Pilot Duration:** December 2019 to June 2022 (7 months).

**End-users involved:**

Patients: total 19 (9 male and 10 female, average age of 53.1±12.5 years old);

Physicians: total 2 (2 male).

**End-users feedback data collection methods:** On the last day of the follow-up period, the researcher conducted a phone call-based semi-structured interview to collect the patient’s feedback, considering the interaction with technology and the overall experience.

The pilot initiative 2 characterization resulted in four features (Table 4), which are: (1) outcome collection using a mobile-based chatbot; (2) RPM-based therapy management using chatbot; (3) surgical team alert email notification; and (4) instant data availability. The PAR team evaluated the second and fourth features providing the most value for the end-users (physicians) since the physician feedback was very positive. The first feature was assessed as low value as the IoT devices were already selected as the channel to collect the outcomes. In addition, the third feature was also evaluated as low value since physicians and nurses had to assess the patient’s RPM data daily.

**Table 4:** List of features of pilot initiative 2.

Feature (originator)	Utilization feedback	HP feedback (N)	Patient feedback (N)	Evaluation
1 - Outcome collection using a mobile-based chatbot (Physicians)	Total of questionnaires replied (231); Total number of questionnaires replied on average per patient (12.2)	Good, but found some problems/challenges (6)	Good, but found some problems/challenges (5); Did not like it or could not use it, due to problems (1); Patient totally agreed that he/she felt well supported with this service (7); Patient totally agreed that the service interferes with patient’s daily routine (3); Patient totally agreed that the service should be recommended to people with a health condition similar to his/her (8); Patient totally agreed that he/she was satisfied with this service (8)	Major changes, high effort, low value
2 - RPM-based therapy management using chatbot (Physicians; Developers)	Total prescriptions (206); Total prescriptions on average per patient (10.8); Total of questionnaires requested (239); Total number of questionnaires requested on average per patient (12.6)	Good, but found some problems/challenges (7); Good, did not find problems/challenges (2)	No feedback recorded	Minor changes, low effort, high value
3 - Surgical team alert email notification (Developers)	Total of emails generated (231);Total number of emails generated on average per patient (12.2)	Good, did not find problems/challenges (1)	No feedback recorded	Minor changes, low effort, low value
4 - Instant data availability (Developers)	Median time elapsed between the question and the answer was 12 min	Good, did not find problems/challenges (1)	No feedback recorded	Major changes, high effort, high value

The third pilot initiative refers to an RPM follow-up pilot service conducted by one of the partners of the PAR team in the scope of the COVID-19 pandemic (Box 3). The main similarity between the pilot initiative and the funded project was the isolation context of the target population during follow-up. The second similarity consists of adapting existing technology to the needs of an elderly population, maximizing user interaction and experience.

Box 3. **Pilot Initiative 3: NursingHomeRPM: Remote care delivery in nursing homes (38)**

**Motivation:** Portugal 2020 funded research and development projects for testing and optimization of technological infrastructures in the context of COVID-19.

**Goal:** Digitize the care provided in a nursing home during the COVID-19 pandemic with each partner’s existing technology.

**Deployment Site:** Private nursing home, Cascais, Portugal.

**Population:** Nursing home residents.

**Previous Intervention:** The standard care provided in the nursing home required formal caregivers to register physical needs, including personal hygiene or grooming, dressing, toileting, transferring or ambulating, and eating in a notebook.

**New Intervention:** The digital transformation consisted of developing and implementing a mobile application that could connect with IoT devices to monitor its residents.

**RPM Period:** 30 days.

**RPM Outcomes Measurements:** seven in total - blood pressure, temperature, blood oxygen levels, blood glucose levels, daily mood tracker, ability to conduct activities of daily living, and quality of life.

**Responsible Team:**

Nursing Home Professionals: total 1 (1 male, age of 45 years old);

Physicians: total 1 (1 female, age of 45 years old);

Developers: total 8 (3 female and 5 male, average age of 34±8.9 years old);

Researchers: total 3 (2 female and 1 male, average age of 32.3±10.7 years old) with expertise in digital health services design, digital and cost analysis;

User Interaction/User Experience (UX/UI) designer: total 1 (1 female, age of 30 years old).

**The technology used:** The developers proposed an existing IoT kit to be used in the nursing home to collect outcomes, which includes an oximeter, blood pressure monitor, thermometer, glucometer, and an Android tablet. A mobile application allowed nursing home staff to report outcomes through several (Yes/No) questions survey and 4-point Likert scale questions, automatically collecting clinical parameters from the IoT devices.

**Pilot Activities:** The team worked together to create an RPM platform with multiple user roles (manager, physician, nurse, informal caregiver, and patient) to collect patient outcomes.

**Pilot Duration:** June to November 2020 (5 months).

**End-users involved:**

Patients: total 10 (5 male and 5 female with an average age of 81.0±8.0 years old);

Physician: total 1 (1 female);

Nurses: total 1 (1 male);

Nursing Home: 1 director (male), 6 staff (6 female).

**End-users feedback data collection methods:** User experience and interaction testing sessions were conducted with the nursing home director, one staff member, and one nursing home resident.

The pilot initiative 3 characterization resulted in four features (Table 5), which are: (1) outcome collection using a mobile app connected to IoT devices; (2) RPM-based therapy management; (3) interoperability using FHIR; and (4) role definition. The PAR team evaluated the first three providing the most value for end-users (Nursing Home director and physicians) since the director and physician considered as a must-have requirement. Although the fourth feature was considered to provide moderate value, the PAR team considered that it required significant changes and high effort to develop.

**Table 5:** List of features of pilot initiative 3.

Feature (originator)	Utilization feedback	NH quotes	Evaluation
1 - Outcome collection using a mobile app connected to IoT devices (Developers)	The total percentage of questionnaires replied was 35.1%.	Nursing Home Director: “does not recommend presenting past data, senior is interested in how it is now. The “Start” button comment could be larger and colored green “Nursing Home Director: “add interaction with smileys when the quiz is completed/gamification.” Nursing Home Director and Physician: “To avoid errors in the measurement of signals, the Protocol should be the same for all Users, that is, all sensors must be used by all Users that will participate in the pilot, except those referring to methods invasive, for example capillary blood glucose, which should be exclusive to Patients with Diabetes.”	Minor changes, low effort, high value
2 - RPM-based therapy management (Nursing Home Director)	No metric recorded	Nursing Home Director: “The most difficult thing for us is to manage the medication prescribed by the physician. During this time (COVID) the physician calls us and reviews the medication prescribed to all seniors. What I do is to write it down on a table and then I put the paper on the wall so the staff don’t forget to give the right medication to each senior.”	Minor changes, low effort, high value
3 - Interoperability using FHIR (Developer)	No metric recorded	No feedback recorded	Major changes, high effort, high value
4 - Role definition (Nursing Home Director)	No metric recorded	Nursing Home Director: “Each assistant must end their session as the tablet is shared.”	Major changes, high effort, moderate value

### 3.2. Reflection phase

This phase involved five meetings with an average participation of eight PAR team members and an average of 42 min (3 h and 30 min in total).

#### 3.2.1. Pilot initiative participatory assessment and features prioritization

**Figure 2:**
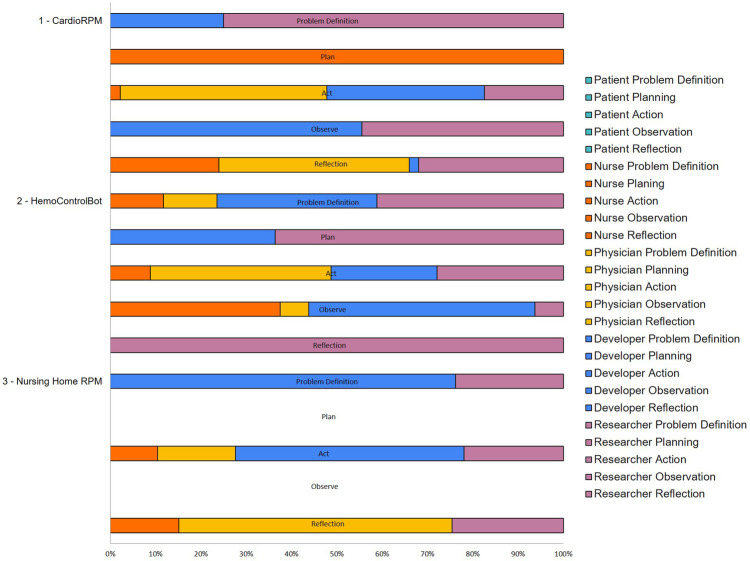
Level of participation of each team role per PAR phase per pilot initiative.

An overview of Figure 2 reveals that the participation level according to each group was Developers (33.1%), Researchers (31.5%), Physicians (17.1%), Nurses (15.0%), and Patients (3.2%). The Researchers played the leading role in the Problem Definition and Planning phases (73.9% and 58.3%, respectively). In the Action and Observation phases, the Researchers group was the most participating group (34.3% and 53.9%, respectively). Finally, in the Reflection phase, the most participating role was the Nurses (34.2%). A more detailed analysis of each pilot initiative informs that the Researchers participated more in the first pilot initiative (39.7%), Developers and Physicians share the first position in the second pilot initiative (29.4%), and the Developers in the third pilot initiative (45.8%). In contrast, the patients were only present, with minor participation, in the first and third pilot initiatives (5.5% and 4.5%, respectively). In the first pilot initiative, the Reflection phase was the phase with the highest participation roles diversity, and the Planning phase was the lowest. In the second pilot initiative, the Problem Definition, Action, and Observation were the phases with the highest participation roles diversity. Finally, the third pilot initiative had no participation in Planning and Observation phases and the highest participation role diversity in the Action phase.

**Figure 3:**
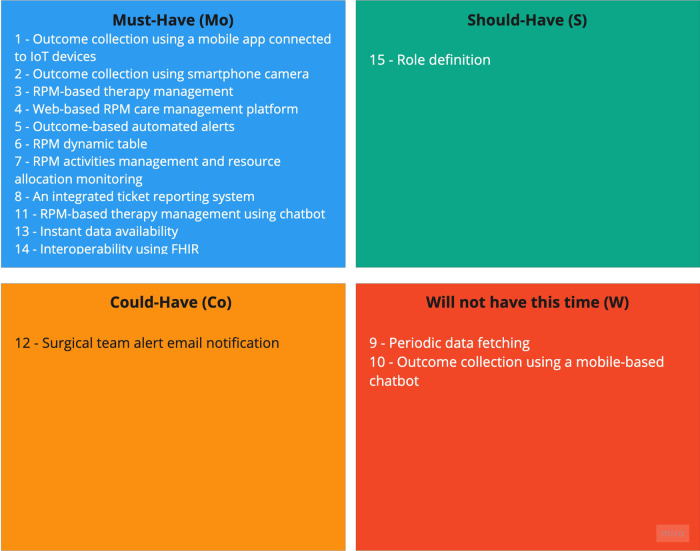
Diagram of the features of the three previously developed pilot initiatives. Each category is represented with a letter and a color: must-have - Mo (blue), should-have - S (green), could-have Co (yellow), and will not have this time - W (red).

From the three pilot initiatives the PAR team extracted 15 features and classified 11 as must-haves. Figure 3 illustrates the feature prioritization for development. All the features classified with high value for the end-users were prioritized as Must-Have on the enhanced version of the RPM-platform. The feature of Role definition was prioritized as Should-Have because although it was evaluated as a moderate-value feature, it implied significant changes and, consequently, high effort to implement. In contrast, the email notification feature was prioritized as Could-Have because although it imposes lower changes and minor effort, it was evaluated to bring low value to the end users.

#### 3.2.2. Evaluation of the PAR approach

**Figure 4:**
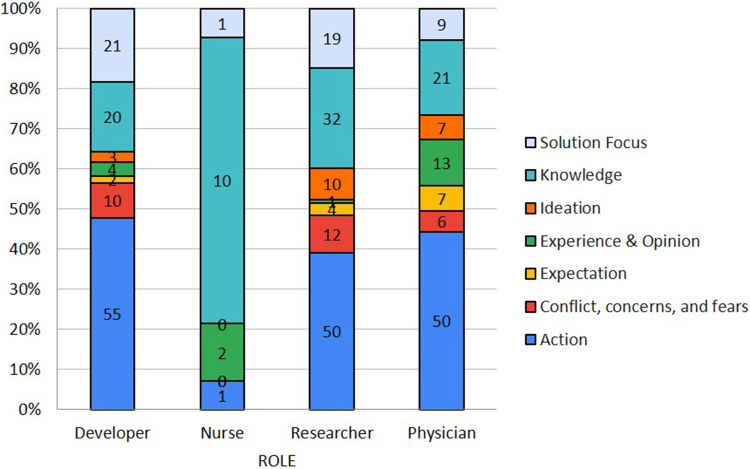
Different contributions according to PAR team role.

**Figure 5:**
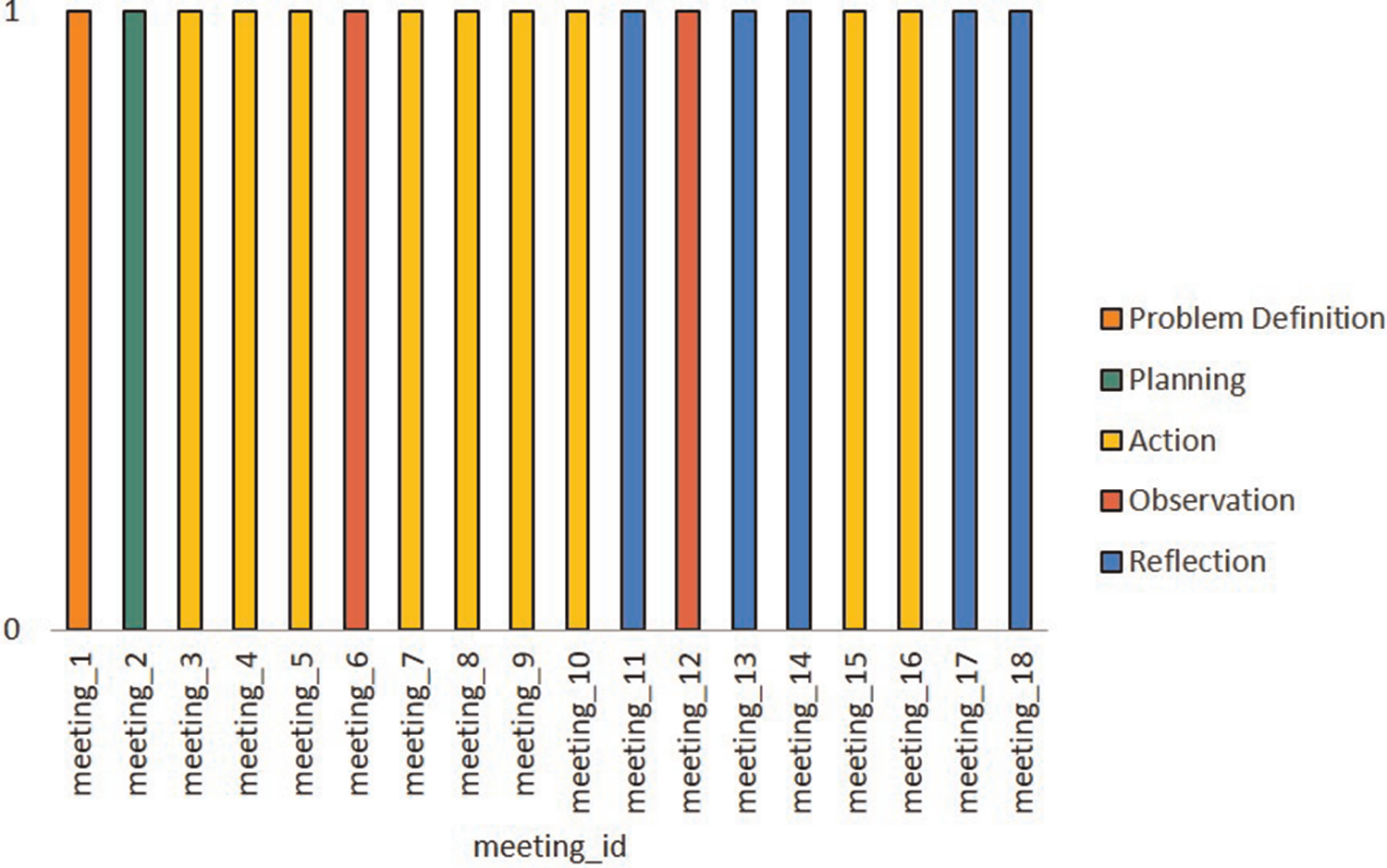
Distribution of meetings per phase.

The top three topics most covered across the phases were Design (39.7%), Development (22.2%), and Execution and Implementation (18.5%). The top three meeting focus were Data Analysis Framework Requirements (23.4%), Study Protocol Writing (16.6%), Modelling and Simulation (15.6%). The top three meeting outcomes contributions were: Improved research and development system (35.0%), Better characterization and understanding of the complex socio-technical-economic constraints to sustainable software development production and care service provision (25.2%), and Better understanding, on the part of researchers, of systems used by healthcare professionals and patients (22.0%). The participation level according to each role in the meetings was: Researchers (52.9%), Developers (47.5%), Physicians (46.7%), and Nurses (5.8%). The top three contributions of the participants involve suggesting or planning actions (42.2%), sharing or generating knowledge (22.4%), and discussing the solution (13.5%). Figure 4 exposes the most frequent type of contribution by each PAR team role. Physicians, Researchers, and Developers suggest and plan more actions (44.2%, 39.1%, and 47.8%, respectively), while Nurses are more dedicated to share and generate knowledge (71.4%). The participation level according to of each role in the meetings was: Researchers (52.9%), Developers (47.5%), Physicians (46.7%), and Nurses (5.8%). The approach involved two action-reflection cycles illustrated in Figure 5. In this figure, it is also illustrated the distribution of the meetings per phase to define the final list of features and prioritize the development. Most of the meetings were dedicated to the Action phase (9 meetings), followed by the Reflection Phase (5 meetings), Observation (2 meetings), and Problem Definition (1 meeting) and Planning (1 meeting).

## 4. Discussion

The proposed RPM-based platform (Supplementary Material 5) is the result of a PAR approach and comprises features suggested, tested, and evaluated by different relevant stakeholders, from end-users, such as patients and healthcare professionals, to software developers and researchers. These features make the platform more robust and ready to integrate value assessment and AI-based decision support tools to increase access to RPM surgical follow-up care in pandemic contexts.

The PAR project was initiated by a consortium of partners, funded by a Portuguese national research agency, that aimed to ensure that the following cycle of technological development would allow the provision of care to a larger and diversified sample of patients in a public hospital. Therefore, patients and healthcare professionals had to be part of the research process so that their needs were not compromised at the expense of scalability (39). Problem definition and context characterization are two important domains usually neglected by researchers in scalability assessment (40). The most important features for the patients were the outcomes collection using a mobile application, smartphone camera, and IoT devices, outcome-based automated alerts, and the web-based RPM care management platform. Patients refer to these features by highlighting how they changed their daily routines to provide information to physicians and nurses and how the latter called them every time the outcome values were not the ones expected. In addition to the patient-preferred features, physicians also showed a preference for the feature of RPM dynamic table and RPM-based therapy management using a chatbot. This is explained by the healthcare professionals’ need to act quickly on generated alerts, automatize some of their actions, and provide personalized care accordingly. In contrast, developers and researchers showed a preference for backend features. On the one hand, developers suggested features related to interoperability using standard data formats and instant data availability. On the other hand, researchers suggested features that would support their research, such as concerning the feature of RPM activities management and resource allocation monitoring (41).

The PAR approach allowed the aggregation of different stakeholders’ opinions of the problem, guaranteeing their engagement throughout the project and the general interest of the research. As discussed by experts in digital health the interdisciplinary co-creation is an enabler for scaling up digital solutions (41). The flexibility inherent to this approach enabled collaboration among partners to select the final set of features by sharing previously conducted work, experience, and acquired knowledge. Therefore, the proposed RPM solution may have a high agreement with the users’ needs avoiding wasting time and resources, which is particularly relevant in healthcare providing services (42). This RPM platform helps to give more personalized care: the platform provides useful patient information to be used by the clinical team to make patient-centered medical decisions from the collected data. In addition, the high reported levels of adherence concerning some features may indicate that the patients are prone to use these RPM systems to interact and to be followed up by clinical teams. Some patient testimonials revealed they felt they were being followed up closely, conveying a greater sense of (2).

The proposed platform data infrastructure allows data collection considering the patient pathway. Contrary to most hospitals’ information systems (43), this platform collects data associated with the different activities of the patient journey during the intervention. For each activity in the patient pathway, the allocated resources type, quantity, and time are recorded. This information is integrated with the outcomes’ stability analysis allowing the intervention’s value assessment.

The dynamic and iterative nature of the research process allowed the different stakeholders to cover distinct topics from design to execution and implementation and focus, such as data analysis framework requirements and modeling and simulation simultaneously, rather than sequentially, as in other research approaches. This work also emphasized how the information flows and is exchanged among physicians, nurses, researchers, and developers, revealing that all should be in the different phases of software development cycles.

This work has some limitations considering the implementation of the PAR approach since the patients were only part of the research process indirectly through feedback evaluation in two of the three case studies. This limitation was caused by the COVID-19 context that excluded the option of group meetings with the patients. Another limitation was the lack of observations considering the topics that generated more or less agreement during meetings. This would allow a better analysis of leadership dominant and oppressive roles in the PAR team (41). The PAR approach encourages the researchers to focus on the practitioners’ problems and work collaboratively on solutions to those problems (15); therefore, another limitation might be related to the generalizability of the RPM platform to other contexts. Two significant limitations of the proposed RPM-based platform are, first, the lack of integration with the hospital’s information system. Second, the limited sample of patients that tested each feature. However, the PAR team is already running a clinical study with 150 patients.

Future work should focus on the evaluation of the implemented value assessment and AI-based decision-support tools’ impact on the clinical practice to increase access to high-quality RPM-based surgical follow-up services. outcomes’ stability analysis allowing the intervention’s value assessment.

## 5. Conclusion

RPM-based follow-up services were highly adopted during the pandemic, driving healthcare organizations to scale-up ongoing pilot initiatives. The scalability of RPM services must consider contextual factors, such as individuals’ and organizations’ interests and needs, that influence its uptake into routine use. The PAR approach allowed to simultaneously design, develop, test, and evaluate the RPM platform features with the contribution of patients, healthcare professionals, developers, and researchers. Participatory research is needed to scale up RPM technologies into widespread clinical routine usage.

## Data Availability

The datasets generated during and/or analysed during the current study are available from the corresponding author on reasonable request. Requests to access the datasets should be directed to *ana.londral@vohcolab.org*.
