# Supplementary material for: Scaling-up digital follow-up care services: collaborative development and implementation of Remote Patient Monitoring pilot initiatives to increase access to follow-up care
Source: Front Digit Health. 2022 Dec 7;4:1006447. doi: 10.3389/fdgth.2022.1006447 (PMC9768029; doi:10.3389/fdgth.2022.1006447)

Supplementary Material

1 Supplementary Material 1 - Minutes and reports coding scheme

Table 1 (T1) - Minutes and reports coding scheme

TOPIC	FOCUS	INTERACTION	REACTION	OUTCOME	AGENT
Project Planning	Project presentation	Highlighted a conflict	All showed that they agreed with the statement.	Generation of new technological feature	Researcher
Project Management	Problem Definition	Shared a concern or fear	Most showed that they agreed with the statement.	Adoption of new technological feature	Nurse (end-user)
Search	Objectives Definition & Results Anticipation	Shared an opinion	Half of the participants showed that they agreed with the statement.	Increased democratization	Developer
Design	Roadmap Definition	Shared an expectation	Almost no one showed that they agreed with the statement.	Increased cost-effectiveness	Surgeon (end-user)
Development	Budget Definition	Shared an experience	No one showed that they agreed with the statement.	Better understanding, on the part of researchers, of systems used by healthcare professionals/patients	
Execution & Implementation	Team Definition	Solution-related question	No further discussion required.	Better characterization and understanding the complex bio-physical and socio-economic constraints to sustainable software development production and care service provision	
Evaluation & Assessment	Data Management Plan	Shared Knowledge	Reaction not recorded.	Development of technologies that cope with user needs	
	Team Changes	Solution-related problem		Improved research and extension system	
	Roadmap Execution	Shared an idea		Empowerment by improving healthcare professionals' capacity for self-directed technology development and ability to adapt	

				healthcare systems to changing conditions	
	Budget Execution	Selected an idea		No outcome is expected	
	Literature Review	Suggested an action			
	Benchmarking	Planned an action			
	Hospital Visits	Generated Knowledge			
	Technological Solution Requirements				
	Data Analysis Frameworks Requirements				
	Data Storage and Protection Requirements				
	Literacy Activities Requirements				
	Study Protocol Writing				
	Software Development				
	Modelling & Simulation				
	Data Storage and Protection Development				
	Literacy Activities Development				
	Study Protocol Approval				
	Technology Trial				
	Data Collection & Processing				
	Data Storage and Protection Testing				
	Literacy Activities Testing				
	Technology Evaluation				
	Data Analysis & Interpretation				
	Data Storage and Protection Evaluation				
	Literacy Activities Evaluation				
	Results communication & Dissemination				

2 Supplementary Material 2 - Semi-structured interviews coding scheme

Table 2 (T2) - Semi-structured interviews coding scheme

FEEDBACK ON EXISTING FEATURES	IMPROVEMENTS AND NEW IDEIAS	GENERATED LEARNING	PROOF OF ACCEPTANCE	REFLECTIONS
Instructions	Telehealth Video Consultation	Learned how to read and control the outcomes measurements	Patient/Caregiver wants to use one or more IoT devices as much as they can	Reflection on the past
RPM period	Add a new patient-reported outcome measurement	Searched more about RPM-based service and learned about its impact	Patient and/or caregiver showed the desire to keep one or more IoT devices	Expectations for the future
Outcome collection using IoT devices	Outcome collection using voice-based message	Learned more about his/her health and how to improve habits	Patient/caregiver share that measuring the outcomes were part of his/her daily routine	Beliefs
Outcome collection using mobile app questionnaires	Give outcome analysis reports to patients and caregivers			Preferences
Outcome collection using smartphone camera	Other			Patient/Caregiver recommends the RPM service to other patients
Outcome collection using a chatbot				Patient/Caregiver reflects about the type of patients that should have access to RPM-based services
RPM-based therapy management				Patient/Caregiver reflects about the barriers to other patients to access to RPM-based services
RPM-based therapy management using a chatbot				
Web-based RPM care management platform				
Role definition				
Surgical team alert email notification				
Outcome-based automated alerts				
Patient remote monitoring dynamic table				

RPM activities management and resource allocation monitoring				
Integrated ticket reporting system				
Periodic data fetching				
Instant data availability				
Interoperability using FHIR				
Satisfaction & Experience Questionnaire				

3 Supplementary Material 3 - Root causes of the defined problem

Supplementary Figure 1 (F1) - The Ishikawa diagram of the root causes of the defined problem.

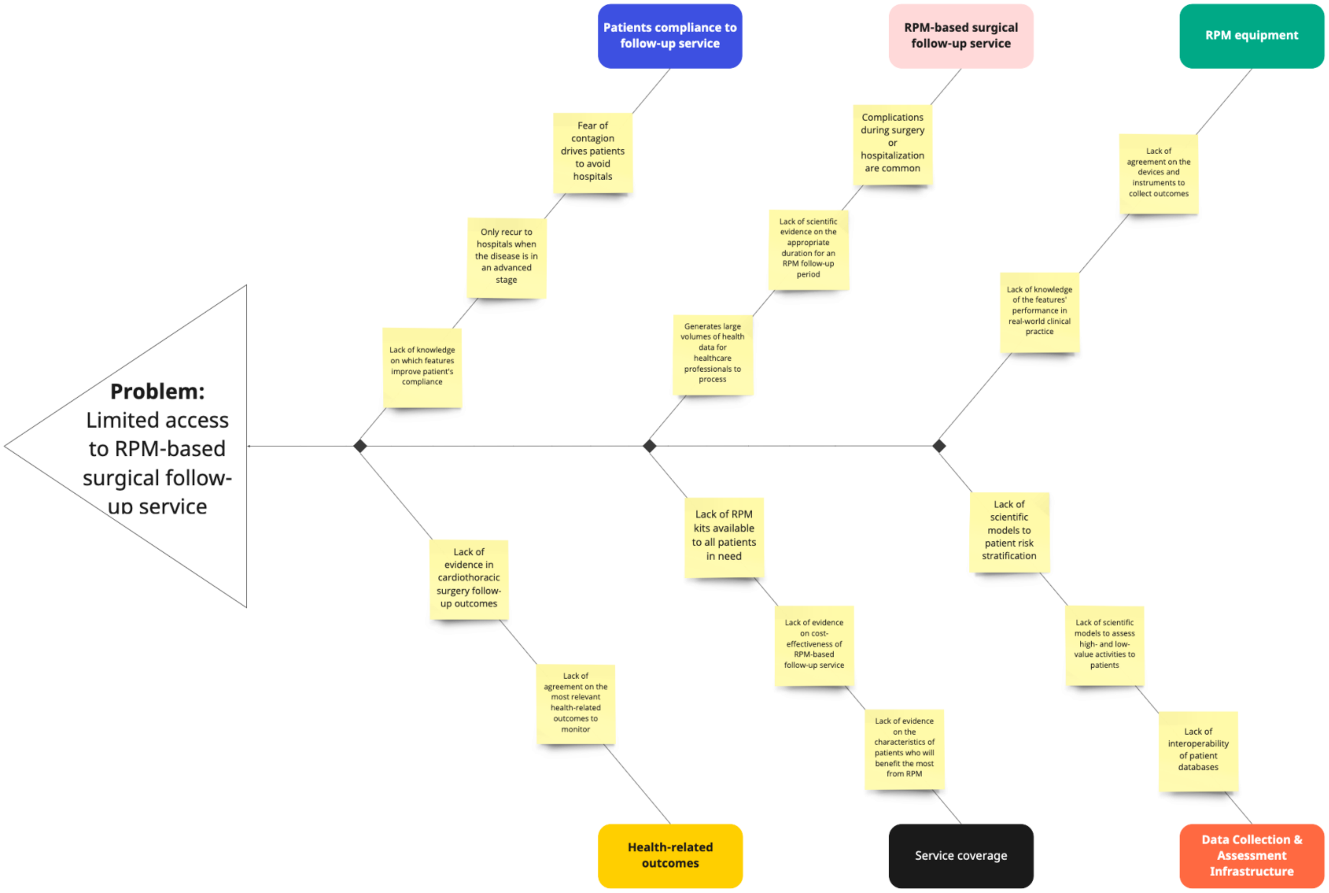

4 Supplementary Material 4 - Root causes of the defined problem

Supplementary Figure 2 (F2) - Pilot initiative iterative development approach

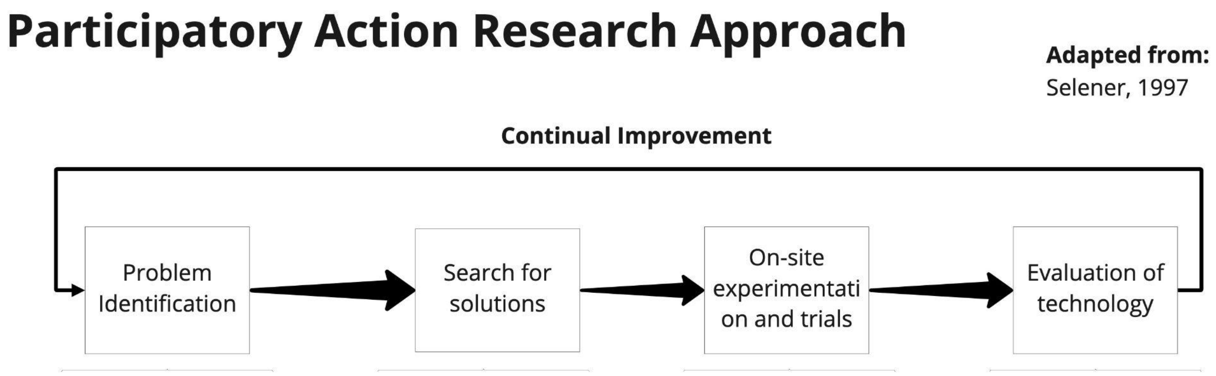

5 Supplementary Material 5 - Web platform and smartphone application description

The RPM solution includes one Android smartphone application connected to IoT devices and one web-based application.

1. Web platform

The aforementioned features were integrated to create a new platform for RPM. This integration was made in a modular way, so the implemented features can be reused, and new ones can be added in the future. Below, some figures depict features of the proposed platform: a list of remotely monitored patients, medication list, monitoring action, list of photos of the wound, and outcomes analysis graphics.

Figure 3 depicts the RPM dynamic table feature, which consists of list of monitored patients and their respective outcomes. Each line has information regarding a patient. The first icon on the left has the patient ID. The following group of icons represent the last measured value of each outcome. If the value is outside of the normal range, the icon has a different color than grey. This way, the surgical team can quickly assess the patients that need more attention. If the icon is faded, the represented value is not from that day. Behind the big grey the rectangle is the name of the patient. On the right side, it is possible to observe a bell icon that lights up if a monitoring action was taken during that day and a view button that leads to the detailed page of that patient.

Supplementary Figure 3 (F3) - RPM dynamic table feature

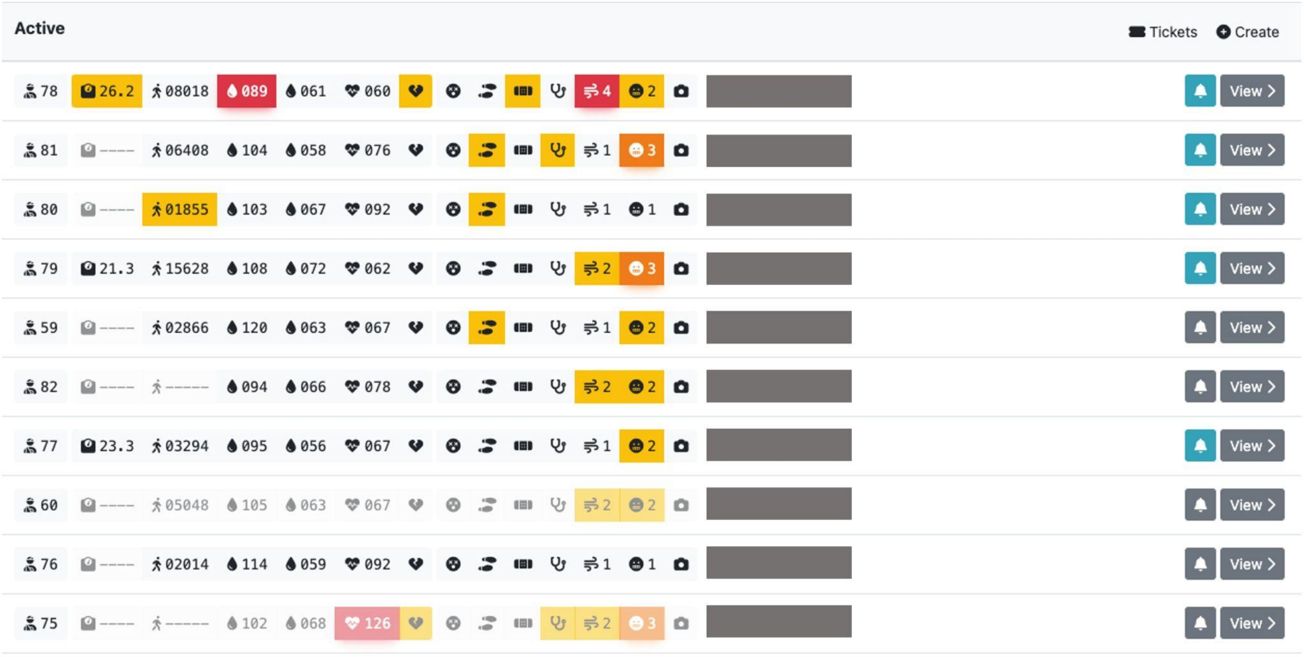

In figure 4, it is possible to observe an example of the application of the RPM-based therapy management feature, which consists of a patient's medication list. In addition to information related to taking the medication and its type, it is also possible to access notes that have been added for both the patient and the clinical team. Also, the clinical team can manage each medication the patient takes.

Supplementary Figure 4 (F4) - RPM-based therapy management feature

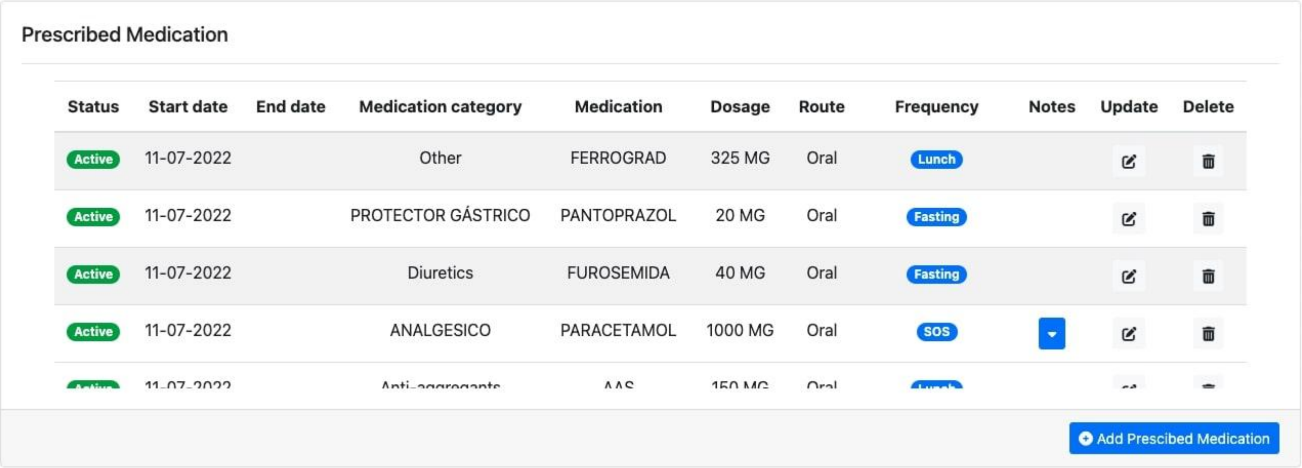

Prescribed Medication

Status	Start date	End date	Medication category	Medication	Dosage	Route	Frequency	Notes	Update	Delete
Active	11-07-2022		Other	FERROGRAD	325 MG	Oral	Lunch			
Active	11-07-2022		PROTECTOR GÁSTRICO	PANTOPRAZOL	20 MG	Oral	Fasting			
Active	11-07-2022		Diuretics	FUROSEMIDA	40 MG	Oral	Fasting			
Active	11-07-2022		ANALGESICO	PARACETAMOL	1000 MG	Oral	SOS			
[illegible]	11-07-2022		Anti-aggregants	AAS	150 MG	Oral	[illegible]			

Add Prescibed Medication

The feature related to RPM activities management and allocation of resources is illustrated in Figure 5. Monitoring actions are displayed in a vertical timeline where at the top are the most recent. This figure can be divided into four elements: target symptom, action activities (such as phone calls or hospital appointments), action types (such as medication adjustment or health literacy reinforcement), and action notes. The first one is associated with the reported outcomes that lead to that action. The second one is related to the activity that was performed. The third one corresponds to what was done during the activity. The last one shows the notes the clinical team member wrote associated with that action. Creating well-adapted and detailed clinical actions is key to identifying the added value of different clinical actions within an RPM service.

Supplementary Figure 5 (F5) - RPM activities management and allocation of resources

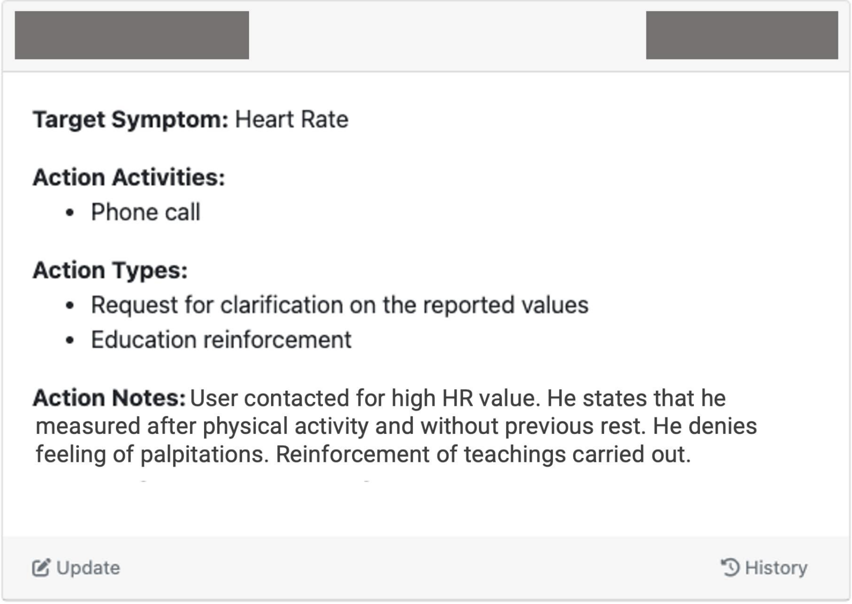

Figure 6 shows the web platform UI that enables the visualization of PROMs evolution through time. The (A) part shows an example of the list of surgical wound pictures sent by the patient during the RPM period. The web platform allowed the surgical team to evaluate the picture regarding its quality and healing changes in the surgical wound. The (B) part shows an example of a graphic of the collected outcomes, where it is possible to check if the reported values are abnormal.

Supplementary Figure 6 (F6) - web platform UI that enables the visualization of PROMs evolution through time

A **B**

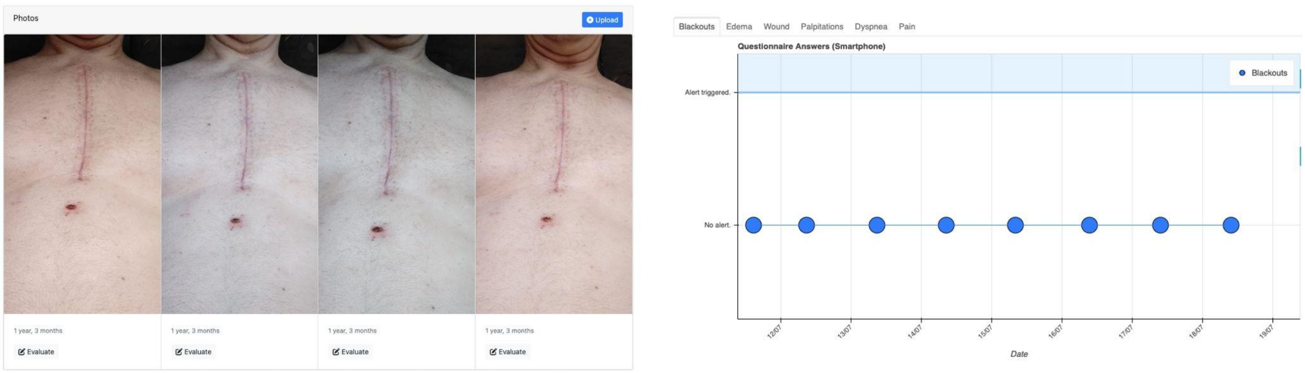

2. **<u>Smartphone Application</u>**

The smartphone app (Figure 7) is provided to the patient together with a set of IoT devices, which includes a weight scale (A\&D Medical UA-352BLE), a blood pressure monitor (A\&D Medical UA-651BLE), a smartwatch (Shenzhen E80), and a smartphone (Xiamoi Mi A2 Lite). The IoT kit allows the collection of the following measures: weight, diastolic and systolic blood pressure, heart rate at rest, heart rate irregularity, continuous heart rate, and steps. This health parameter collection is executed through the mobile app using Bluetooth.

Within the smartphone app, the patient can explore their health data history (B) and perform additional tasks relevant to the follow-up of these patients (A). These tasks include acquiring pictures, answering questionnaires for self-reported signs and symptoms, and accessing the daily medication plan. The patient is requested by the surgical team to perform the tasks at a specific pre-defined time.

To promote engagement, the app allows patients to receive messages (C). These messages are received via the proposed web app. They can either be pre-defined automated messages, triggered according to the adherence and health specificities of each patient, or written by the clinical team on therapeutic recommendations.

To assure standardized interoperability, the smartphone app connects with FHIR based server. The FHIR incorporation allows a straightforward exchange of health data with multiple health information systems that integrate this standard.

Supplementary Material

Supplementary Figure 7 (F7) - Smartphone Application

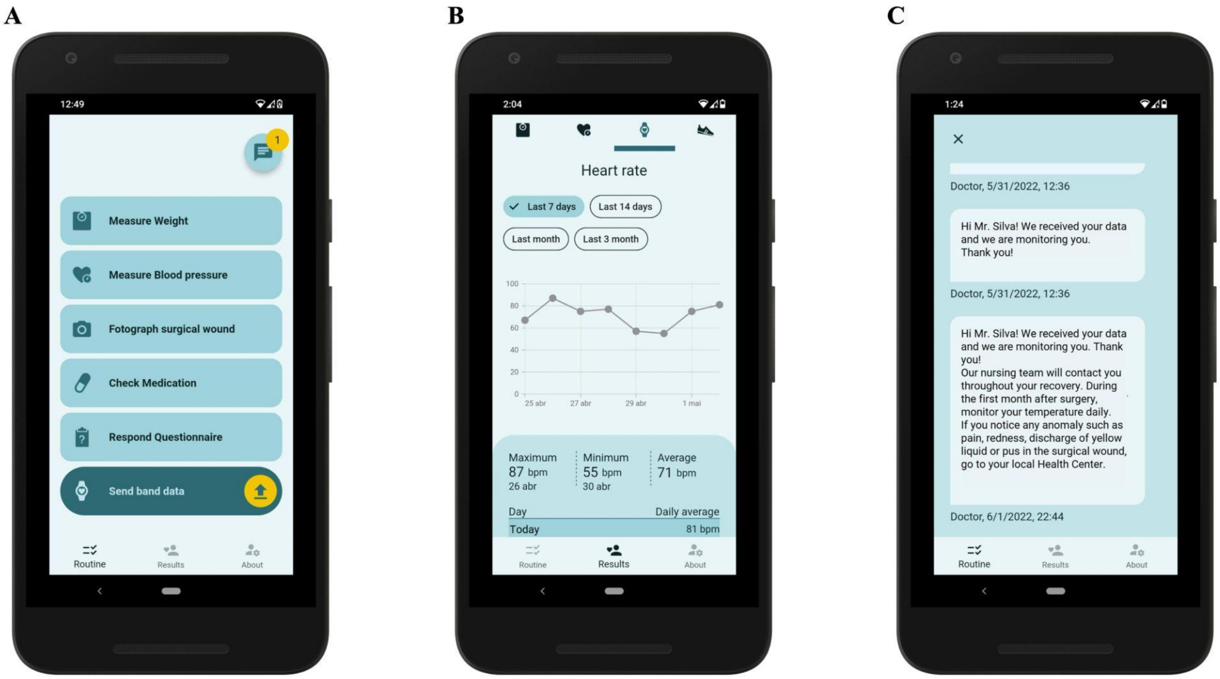

Supplement: Supplementary file 1 [file Datasheet1.pdf]
